# Microwave Assisted Synthesis of Some New Fused 1,2,4-Triazines Bearing Thiophene Moieties With Expected Pharmacological Activity

**DOI:** 10.3390/molecules16064937

**Published:** 2011-06-15

**Authors:** Hosam A. Saad, Mohamed M. Youssef, Mosselhi A. Mosselhi

**Affiliations:** 1 Department of Chemistry, Faculty of Science, Taif University, Taif, 21974, Kingdom of Saudi Arabia; 2 Department of Chemistry, Faculty of Science, Zagazig University, Zagazig, 44511, Egypt; 3 Department of Chemistry, Faculty of Science, Cairo University, Cairo, 12613, Egypt

**Keywords:** 1,2,4-triazinone, thiadiazinone, triazinone, triazine carbonitrile, microwave synthesis

## Abstract

Rapid and efficient solvent-free synthesis of 4-amino-3-mercapto-6-[2-(2-thienyl)vinyl]-1,2,4-triazin-5(4*H*)-one **1** under microwave irradiation is described. Some new fused heterobicyclic nitrogen systems such as 1,2,4-triazino[3,4-*b*][1,3,4]thiadiazinones, 1,3,4-thiadiazolo[2,3-*c*][1,2,4]triazinone and pyrazolo[5,1-*c*]-[1,2,4]triazine-7-carbonitrile, have been synthesized by treatment of **1** with bifunctional oxygen and halogen compounds, CS_2_/KOH and malononitrile *via* heterocyclization reactions, in addition to some uncondensed triazines. Structures of the products have been deduced from their elemental analysis and spectral data (IR, ^1^H-NMR, ^13^C-NMR). Select new synthesized compounds were screened as anticancer agents, with some showing activity as cytotoxic agents against different cancer cell lines.

## 1. Introduction

Microwave assisted organic synthesis (MAOS) continues to affect synthetic chemistry significantly by enabling rapid, reproducible and scaleable chemistry development [1,2,3,4,5]. The use of microwave irradiation is an established tool in organic synthesis for achieving better selectivity, rate enhancement and reduction of thermal degradation byproducts [6,7]. Moreover it is an acknowledged quick alternative and green synthetic organic chemistry technology that also typically results in easier work-up procedures. However these procedures are practically limited as under the high temperatures produced in a microwave oven solvents create high pressure, which may cause explosions. One of the ways to overcome this problem is the use of organic reagents on solid inorganic supports, which has attracted attention because of enhanced selectivity, milder reaction conditions and associated ease of manipulation [8,9]. It also provides an opportunity to work with open vessels and enhances the option of scaling up reactions [10,11].

The biological activities of 1,2,4-triazines have attracted the attention of many chemists because numerous 1,2,4-triazines are biologically active [12,13,14,15,16,17] and are used in medicine, especially as anti AIDS agents, anticancer agents [18,19], antitubercular agents [20], potent CRF receptor antagonists [21], cathepsin K inhibitors [22], and for their anti-anxiety and anti-inflammatory activities [23,24], as well as in agriculture [25,26,27,28]. They also form complexes with metal ions which are used for metal determination. This interest is reinforced by the development of new drugs (e.g., the effective anticonvulsant lamotrigine and anticancer drug tirapazamine), luminescent materials, dyes, specific ligands for complexation with metals, and other compounds based on 1,2,4-triazines.

Fused 1,2,4-triazine systems have also attracted considerable interest in their biological activity. For example 1,2,4-triazolo[5,1-c][1,2,4]triazinones and their sodium salts, along with azoloannelated 1,2,4-triazines, express high activity against different kinds of viruses, including influenza and bird flu (culture H5N1) [29,30,31], and several pyrrolotriazine derivatives were identified as potentially active anticancer agents acting on vascular endothelial growth factor receptor (VEGFR) tyrosine kinases [32].

On the other hand, thiophene-containing compounds are also well known to exhibit various biological effects as BACE1 inhibitors [33], anti-HIV PR inhibitors [34], anti-breast cancer [35], anti-inflammatory [36,37,38], anti-protozoal [39] or antitumor agents [40], potent inhibitors of Pfmrk [41], antitubercular with antimycobacterial activity [42] and inhibitors of EGF-RTK (epidermal growth factor receptor tyrosine kinase) [43].

In light of this we planned to synthesize a series of new 1,2,4-triazines carrying thiophene moieties in the hope of obtaining new products of superior biological activity such as anticancer activity. 

## 2. Results and Discussion

**Scheme 1:**
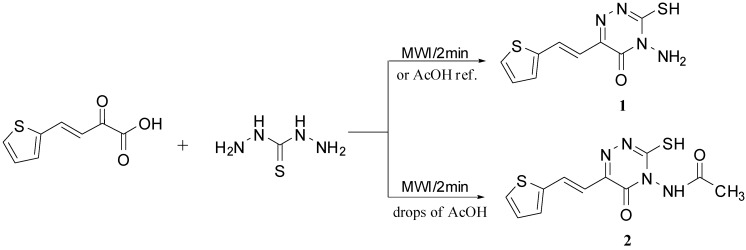
Microwave synthesis of compound **1**.

Attempts were first made to prepare 4-amino-3-mercapto-6-[2-(2-thienyl)vinyl]-1,2,4-triazin-5(4H)-one (**1**) [44] by several routes in order to establish the best method(s) for the preparation of this compound. First, the conventional method by refluxing thiocarbohydrazide [45] with 2-oxo-4-(2-thienyl)but-3-enoic acid [46] in glacial acetic acid [47]. Second, carrying out the solvent free reaction between the two above compounds, under microwave irradiation as described in the literature [48]. Third, *via* the second method, but using some drops of glacial acetic acid, under microwave irradiation, to compare the results of the two methods (Scheme 1). In the conventional method the reaction was complete after two hrs of reflux and the yield was 62%, while in case of microwave irradiation the yield was improved to 98% and the reaction was finished in only 2.0 min.

**Scheme 2:**
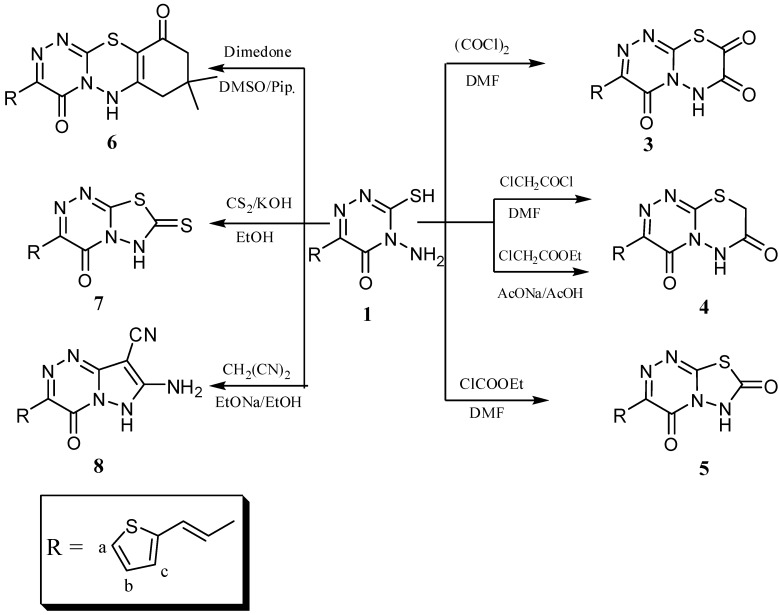
Syntheses of compounds **3**-**8**.

Microwave irradiation of the starting materials in the presence of a few drops of glacial acetic acid yielded 4-(*N*-acetylamino)-3-mercapto-5-oxo-6-[2-(2-thienyl)vinyl]-1,2,4-triazine (**2**). The structures of **1** and **2** were confirmed from their IR, ^1^H-NMR, ^13^C-NMR and elemental analysis data. The ^1^H-NMR of compound **1** showed a singlet at δ 6.51 for NH_2_, two doublets at δ 6.74 and 7.92 ppm for the two *trans* ethylenic protons (H_d_) and (H_e_) with *J* = 15.9-16.0 Hz in addition to the signal of the thiol group at δ 14.00 ppm, respectively. Meanwhile, the ^1^H-NMR of compound **2** showed a singlet at δ 1.91 for CH_3_ and a singlet at 6.51 for NH. Also, the ^13^C-NMR spectrum of **2** showed a CH_3 _peak at δ 21.58. The reactions of **1** with acid chlorides such as oxalyl chloride, chloroacetyl chloride and ethyl chloroformate in DMF yielded [1,2,4]triazino[3,4-*b*][1,3,4]thiadiazine-4,7,8-trione (**3**), [1,2,4]triazino[3,4-*b*][1,3,4]-thiadiazine-4,7(8*H*)-dione (**4**) and [1,3,4]thiadiazolo[2,3-*c*][1,2,4]triazine-4,7(6*H*)-dione (**5**), respectively (Scheme 2). The structure of **3** was confirmed from its IR and ^1^H-NMR data. The IR showed two C=O bands at 1705 and a broad one at 1669 cm^-1^ equivalent to two C=O bands and the ^1^H-NMR showed SH peak and the appearance of new NH peak at δ 12.31 ppm. The IR of **4** showed bands at 3380 for NH and broad at 1665 cm^−1^ equivalent to two C=O bands and the ^1^H-NMR showed no SH peak and a new CH_2 _peak at δ 3.82 ppm. The structure of compound **5** was confirmed from its full analysis [Experimental part]. Compound **6** was formed by cycloaddition of compound **1** with dimedone in boiling DMSO with a few drops of piperidine (Scheme 2). We reported earlier a related reaction and its mechanism [49]. The ^1^H-NMR spectrum of **6** showed a signals at δ 1.25 ppm for two CH_3_ groups, at 2.64 and 3.18 for CH_2_ and CH_2_CO groups, respectively, and the ^13^C-NMR showed signals at δ = 26.80 (2CH_3_), 33.50 (C(CH_3_)_2_), 42.80 (C_9_), 53.5 (C_7_). Also, boiling compound **1** with CS_2_ in dil. ethanolic KOH afforded 3-[2-(2-thienyl)vinyl]-7-thioxo-6,7-dihydro-4*H*-[1,3,4]thiadiazolo[2,3-*c*][1,2,4]-triazin-4-one (**7**) (Scheme 2), while the reaction of compound **1** with malononitrile in ethanolic ethoxide afforded 7-amino-4-oxo-3-[2-(2-thienyl)vinyl]-4,6-dihydropyrazolo[5,1-*c*][1,2,4]triazine-8-carbonitrile (**8**) (Scheme 2). The IR spectrum of **8** showed bands at 3290 for NH and a broad one at 2219 cm^−1^ for C≡N. The ^13^C-NMR showed a signal at δ = 118.2 ppm due to the CN group.

**Scheme 3:**
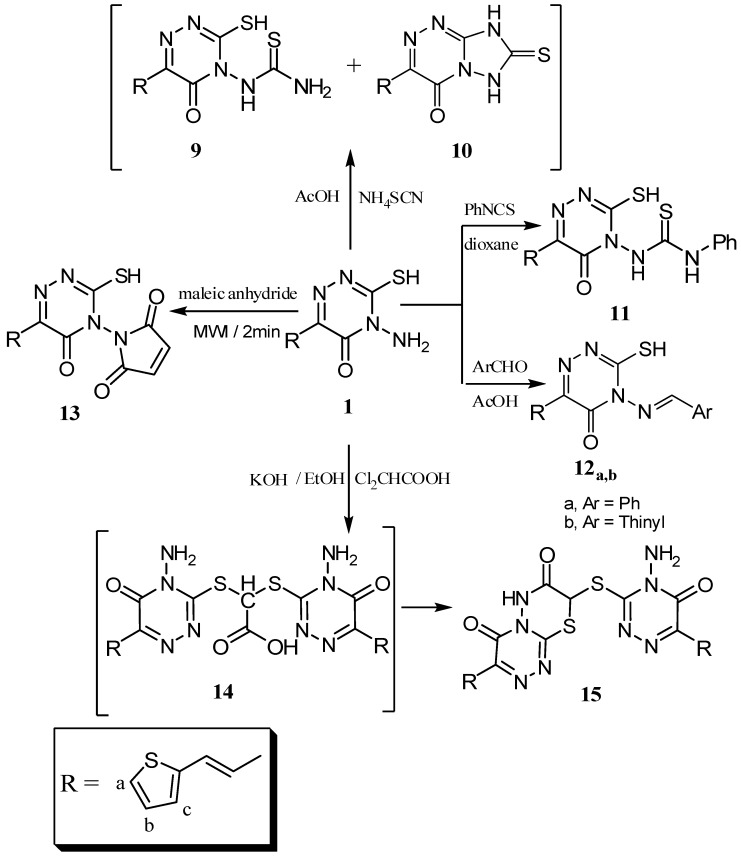
Synthesis of compounds **9**-**15**.

The main objective of the present work was the preparation of fused/isolated heterocyclic nitrogen systems. Thus, addition of ammonium thiocyanate to compound **1** in glacial acetic acid yielded the *N*-substituted thiourea derivatives ***N***-[3-mercapto-5-oxo-6-[2-(2-thienyl)vinyl]-1,2,4-triazin-4(5*H*)-yl]thiourea (**9**) and 2-(2-thienyl)vinyl]-7-thioxo-7,8-dihydro[1,2,4]triazolo[5,1-*c*][1,2,4]triazin-4(6*H*)-one (**10**), respectively (Scheme 3), which, were separated by crystallization. The structures of **9**, **10** were confirmed from their IR, ^1^H-NMR and ^13^C-NMR data and also elemental analysis. The ^1^H-NMR of **9** showed a broad peak at δ = 6.28 ppm due to NH_2_ and two singlets at 12.32 and 14.12 ppm for NH and SH groups, respectively, while the ^1^H-NMR of **10** showed a singlet at δ = 10.27 ppm for NH and no SH peak. Also the reaction of **1** with phenyl isothiocyanate yielded *N*-[3-mercapto-5-oxo-6-[2-(2-thienyl)vinyl]-1,2,4-triazin-4(5*H*)-yl]-*N*'-phenylthio-urea (**11**).

Hoping to expand the biological activity, compound **1** was next condensed with aromatic aldehydes such as benzaldehyde or 2-thiophenaldehyde in EtOH-HCl to give the Schiff base products **12_a_** and **12_b_**, respectively (Scheme 3). The structures of **12_a_** and **12_b_** were confirmed from their spectral data. Thus, the ^1^H-NMR recorded the disappearance of the NH_2_ peak of **1** and the appearance of new peaks due to the benzene and thiophene rings. The reaction of **1** with maleic anhydride under microwave irradiation yielded the *N*-(2,5-dioxopyrrolyl)-1,2,4-triazine derivative **13** (Scheme 3). The structure of **13** was confirmed from its IR, ^1^H-NMR, ^13^C-NMR and elemental analysis. The IR showed a broad band at 1675-1667 cm^−1^ due to three amide C=O groups, while the ^1^H-NMR showed a peak at δ = 7.58 ppm for the pyrrole protons.

The treatment of compound **1** with dichloroacetic acid in ethanolic KOH (10%) gave 8-({4-amino-5-oxo-6-[2-(2-thienyl)vinyl]-4,5-dihydro-1,2,4-triazin-3-yl}thio)-3-[2-(2-thienyl)vinyl]-4*H*,6*H*-[1,2,4]-triazino[3,4-*b*][1,3,4]thiadiazine-4,7(8*H*)-dione (**15**), believed to be formed *via* the intermediate **14**. The structure of compound **15** was confirmed from its elemental analysis and its spectral data as well. The ^1^H-NMR recorded a broad signal at δ = 3.95 ppm due to NH_2_ protons and at 4.00 ppm for SCHCO, while its ^13^C-NMR spectrum showed a signal at δ = 38.2 ppm for SCHCO.

## 3. Pharmacological Studies

### 3.1. Cytotoxicity of the Compounds against Hep-G2 Cells

**Figure 1:**
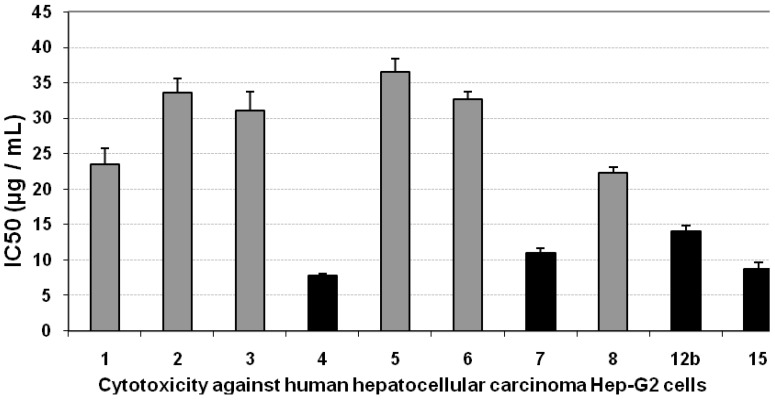
Cytotoxicity (IC50, µg/mL) of different tested compounds against human Hep-G2 cells 48 hours of incubation. The grey bars represent non-cytotoxic compounds, and the black bars represent the promising cytotoxic compounds. Data are representing mean value of IC50 ± SE.

Using the MTT assay we studied the effect of the compounds on the viability of cells after 48 h incubation. Incubation of Hep-G2 cell line with gradually increasing doses of all the compounds led to insignificant changes in the growth of Hep-G2 cells, as indicated from their IC_50_ values (>20 µg/mL), except for compounds **4**, **7**, **11**, **12b** and **15**, which showed inhibition in the viability of Hep-G2 cells compared with the growth of untreated control cells, as concluded from their low IC_50_ values, as indicated by black bars in Figure 1. The positive control, paclitaxol, which is a known anti-cancer drug, resulted in high cytotoxicity against Hep-G2 cells with an IC_50_ value of 643 ng/mL (Figure 1, Table 1).

**Table 1:** Cytotoxicity test using MTT assay against three different human cancer cell lines.

Compd. no.	Mean IC50 (µg/mL)			SE		
	Hep-G2 cells	MCF-7 cells	HCT-116 cells	Hep-G2 cells	MCF-7 cells	HCT-116 cells
**1**	23.51	21.04	32.6	2.25	1.62	1.45
**2**	33.6	31.56	29.01	2.00	2.32	2.18
**3**	31.11	24.5	37.4	2.58	2.15	1.69
**4**	*7.76*	29.11	*4.52*	0.31	0.54	2.01
**5**	36.5	34.73	26.65	1.84	2.52	2.40
**6**	32.6	33.4	29.4	1.13	1.45	2.11
**7**	*11.03*	*18.2*	*8.93*	0.62	0.76	1.26
**11**	22.34	36.41	*11.2*	0.77	1.54	2.51
**12b**	*14.05*	*19.16*	*11.61*	0.80	0.97	1.32
**15**	*8.79*	32.54	*12.67*	0.87	0.61	2.25

### 3.2. Cytotoxicity of the Compounds against MCF-7 Cells

**Figure 2:**
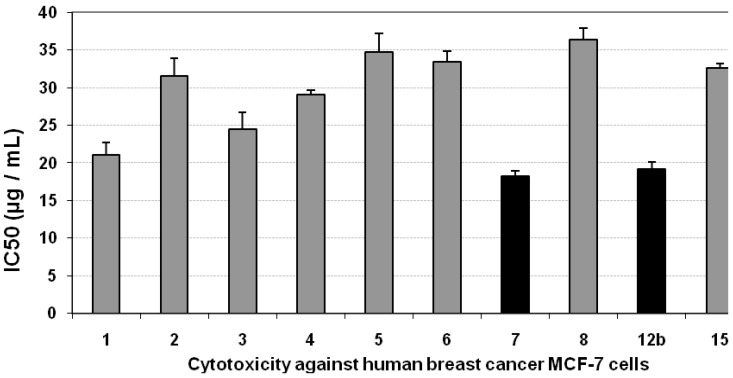
Cytotoxicity (IC_50_, µg/mL) of different tested compounds against human MCF-7 cells 48 hours of incubation. The grey bars represent non-cytotoxic compounds, and the black bars represent the promising cytotoxic compounds. Data are representing mean value of IC_50_ ± SE.

Using the MTT assay we studied the effect of the compounds on the viability of MCF-7 cells after 48 h incubation. Incubation of cell line with most of the tested compounds led to insignificant changes in the growth of MCF-7 cells as indicated from their IC_50_ values (>20 µg/mL), except for compounds **7** and **12b**, which possessed an inhibitory effect on MCF-7 cells viability, compared with the growth of untreated control cells, as concluded from their low IC_50_ values, indicated by black bars in Figure 2. The positive control, paclitaxol, which is a known anti-cancer drug, resulted in high cytotoxicity against MCF-7 cells with an IC_50_ value of 452 ng/mL (Figure 2, Table 1).

### 3.3. Cytotoxicity of the Compounds against HCT-116 Cells

**Figure 3:**
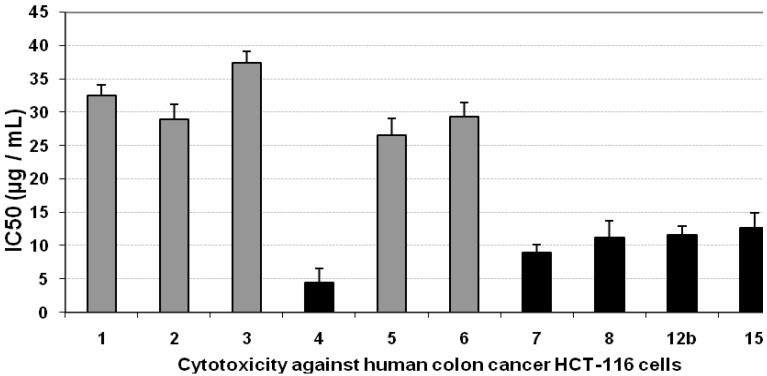
Cytotoxicity (IC50, µg/mL) of different tested compounds against human HCT-116 cells 48 hours of incubation. The grey bars represent non-cytotoxic compounds, and the black bars represent the promising cytotoxic compounds. Data are representing mean value of IC50 ± SE.

The effect of the compounds on the viability of HCT-116 cells after 48 h incubation was studied by the MTT assay. Incubation of HCT-116 cell line with gradually increasing doses of some tested compounds led to insignificant changes in the growth of HCT-116 cells, as indicated from their IC_50_ values (>20 µg/mL). On the other hand, compounds **4**, **7**, **11**, **12b**, and **15** gave a significant inhibition in the viability of HCT-116 cells, compared with the growth of untreated control cells, as concluded from their low IC_50_ values, as iindicated by black bars in Figure 3. The positive control, paclitaxol, which is a known anti-cancer drug, resulted in high cytotoxicity against HCT-116 cells with an IC_50_ value of 709 ng/mL (Figure 3, Table 1).

### 3.4. Percentage of Induced Apoptotic and Necrotic Cells in Hep-G2 Cells

**Figure 4:**
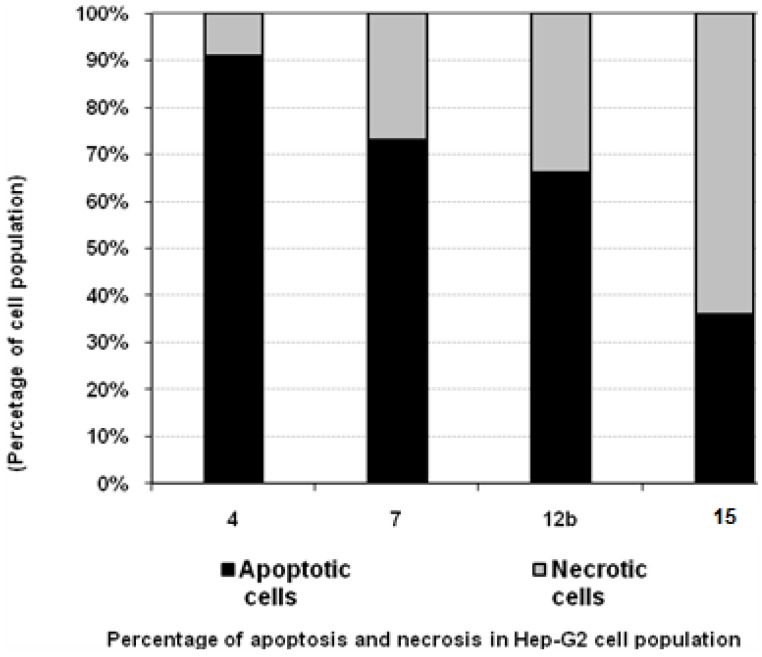
The type of cell death was investigated in Hep-G2 cells after the treatment with the promising cytotoxic compounds, using acridine orange/ethidium bromide staining to compare between the percentage of necrotic cells (grey segment) and the apoptotic cells (black segment). Data are representing mean value ± SE.

According to the findings of the cytotoxicity experiments, compounds **4**, **7** and **12b** possessed a potent cytotoxic effect against Hep-G2 cells. To detect the type of cell death induced in the cells by those compounds, Hep-G2 cells were treated with the IC_50_ values of each compound for 6 h and the apoptosis and necrosis cell population percentages was recorded using acridine orange/ethidium bromide staining. As shown in Figure 4, all of the tested compounds led to an apoptosis-dependant cell death (66-91% of the total dead cell number), while the percentage of necrotic cells was only 9-34% of the total dead cell number, except for compound **15**, which mainly induced necrotic cell death up to 64% (Figure 4, Table 2).

**Table 2:** Apoptosis and necrosis assay for cytotoxic compounds only.

Compd. No.	Hep-G2	
	Apoptotic cells	Necrotic cells
**4**	91	9
**7**	73	27
**12b**	66	34
**15**	36	64

### 3.5. Percentage of Induced Apoptotic and Necrotic Cells in MCF-7 Cells

**Figure 5:**
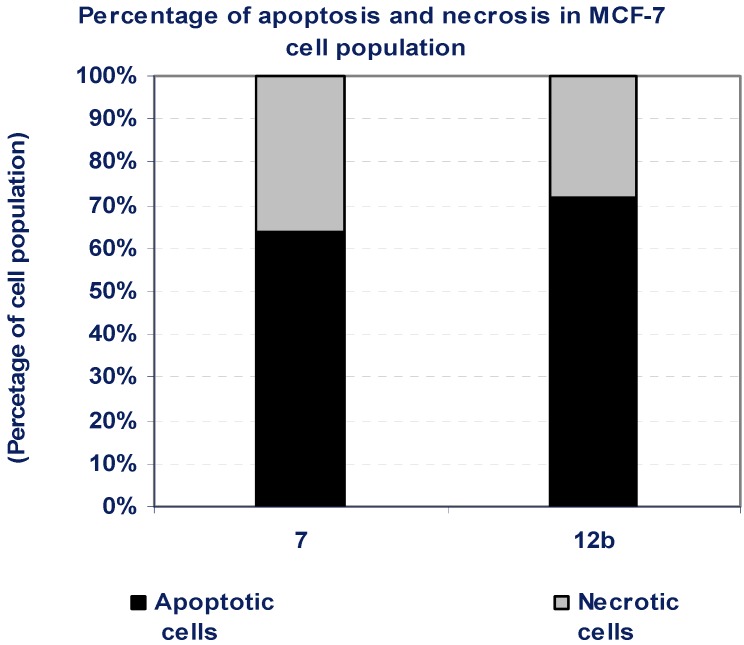
The type of cell death was investigated in MCF-7 cells after the treatment with the promising cytotoxic compounds, using acridine orange/ethidium bromide staining to compare between the percentage of necrotic cells (grey segment) and the apoptotic cells (black segment). Data are representing mean value ± SE.

According to the cytotoxicity experiment results, compounds **7** and **15** possessed a potent cytotoxic effect against MCF-7 cells. To detect the type of cell death induced in the cells by those compounds, MCF-7 cells were treated with the IC_50_ values of each compound for 6 h and the apoptosis and necrosis cell population percentages was recorded using acridine orange/ethidium bromide staining. As shown in Figure 5, both of the tested compounds led mainly to an apoptosis-dependant cell death (64-72% of the total dead cell number), while the percentage of necrotic cells were only 28-36% of the total dead cell number (Figure 5, Table 3).

**Table 3:** Percentage of induced apoptotic and necrotic cells in MCF-7 cells.

Compd. No.	MCF-7	
	Apoptotic cells	Necrotic cells
**7**	64	36
**12b**	72	28

### 3.6. Percentage of Induced Apoptotic and Necrotic Cells in HCT-116 Cells

**Figure 6:**
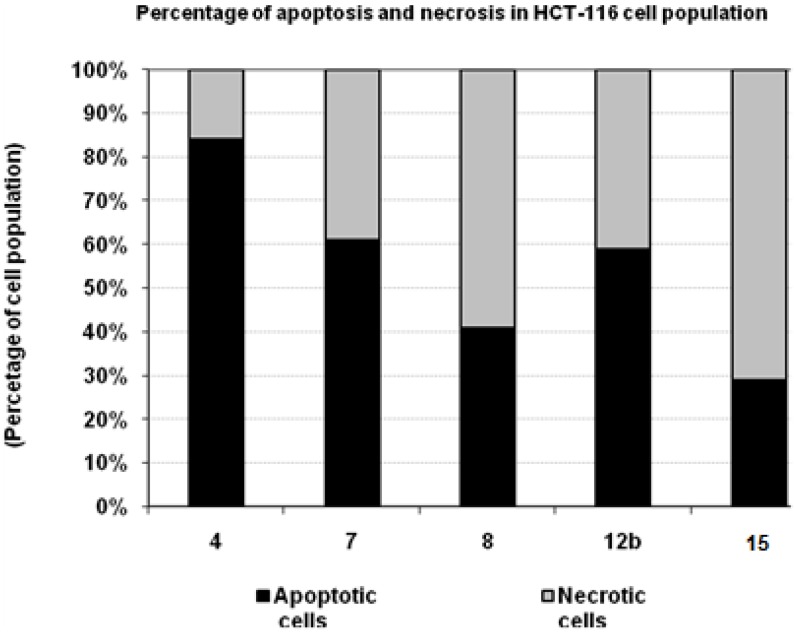
The type of cell death was investigated in HCT-116 cells after the treatment with the promising cytotoxic compounds, using acridine orange/ethidium bromide staining to compare between the percentage of necrotic cells (grey segment) and the apoptotic cells (black segment). Data are representing mean value ± SE.

According to the findings of the cytotoxicity experiments, compounds **4**, **7**, **11**, **12b**, and **15** possessed a potent cytotoxic effect against HCT-116 cells. To detect the type of cell death induced in the cells by those compounds, HCT-116 cells were treated with the IC_50_ values of each compound for 6 h and the apoptosis and necrosis cell population percentages was recorded using acridine orange/ethidium bromide staining. As shown in Figure 6, the tested compounds **4**, and **7** resulted in an apoptosis-dependant cell death (61-84% of the total dead cell number), while compounds **11** and **15** resulted in necrosis-dependant cell death (59-71% of the total dead cell number). On the other hand compound **12b** induced both cell death types (Figure 6, Table 4).

**Table 4:** Percentage of induced apoptotic and necrotic cells in HCT-116 Cells.

Compd. No.	HCT-116	
	Apoptotic cells	Necrotic cells
**4**	84	16
**7**	61	39
**11**	41	59
**12b**	59	41
**15**	29	71

### 3.6. Material and Methods

#### 3.6.1. Cell Culture

Several human cell lines were used in testing the anti-cancer activity including: hepatocellular carcinoma (Hep-G2), colon carcinoma (HCT-116), and histiocytic lymphoma and breast adenocarcinoma (MCF-7) (ATCC, VA, USA). HCT-116 cells were grown in Mc Coy's medium, while all cells were routinely cultured in DMEM (Dulbeco’s Modified Eagle’s Medium) at 37 °C in humidified air containing 5% CO_2_. Media were supplemented with 10% fetal bovine serum (FBS), 2 mM L-glutamine, containing 100 units/mL penicillin G sodium, 100 units/mL streptomycin sulphate, and 250 µg/mL amphotericin B. Monolayer cells were harvested by trypsin/EDTA treatment, while and leukemia cells were harvested by centrifugation. Compound dilutions were tested before assays for endotoxin using Pyrogent^®^ Ultra gel clot assay, and they were found endotoxin free. All experiments were repeated four times, unless mentioned, and the data was represented as (mean ± S.D.). Cell culture material was obtained from Cambrex BioScience (Copenhagen, Denmark), and all chemicals were from Sigma (NY, USA).

#### 3.6.2. Cytotoxicity Assay

Cytotoxicity of tested samples against different types of cells was measured using the MTT Cell Viability Assay. The MTT (3-[4,5-dimethylthiazole-2-yl]-2,5-diphenyltetrazolium bromide) assay is based on the ability of active mitochondrial dehydrogenase enzyme of living cells to cleave the tetrazolium rings of the yellow MTT and form a dark blue insoluble formazan crystals which is largely impermeable to cell membranes, resulting in its accumulation within healthy cells. Solubilization of the cells results in the liberation of crystals, which are then solubilized. The number of viable cells is directly proportional to the level of soluble formazan dark blue color. The extent of the reduction of MTT was quantified by measuring the absorbance at 570 nm [50].

##### 3.6.2.1. Reagent Preparation

MTT solution: 5 mg/mL of MTT in 0.9% NaCl.

Acidified isopropanol: 0.04 N HCl in absolute isopropanol.

##### 3.6.2.2. Procedure

Cells (0.5 × 10^5^ cells/well) in serum-free media were plated in a flat bottom 96-well microplate, and treated with 20 µL of different concentrations of each tested compound for 48 h at 37 °C, in a humidified 5% CO_2_ atmosphere. After incubation, media were removed and 40 µL MTT solution/well were added and incubated for an additional 4 h. MTT crystals were solubilized by adding 180 µL of acidified isopropanol/well and plate was shaken at room temperature, followed by photometric determination of the absorbance at 570 nm using microplate ELISA reader. Triplicate repeats were performed for each concentration and the average was calculated. Data were expressed as the percentage of relative viability compared with the untreated cells compared with the vehicle control, with cytotoxicity indicated by <100% relative viability.

##### 3.6.2.3. Calculations

Percentages of relative viability were calculated using the following equation:

[Absorbance of treated cells / Absorbance of control cells] × 100

Then the half maximal inhibitory concentration IC_50_ was calculated from the equation of the dose response curve.

#### 3.6.3. Apoptosis and Necrosis Staining

The type of cell death was investigated in compound-treated and untreated cells using acridine orange/ethidium bromide staining [51,52]. In brief, cells were treated with the IC_50_ value of each promising compound for 6 h and collected to be treated with acridine orange/ethidium bromide mixture. The vital, necrotic, and apoptotic cells were counted. A mixture of 100 µg/mL acridine orange and 100 µg/mL ethidium bromide was prepared in PBS. The cell uptake of the stain was monitored under a fluorescence microscope, and the apoptotic, necrotic, and viable cells were counted. The early apoptotic cells had yellow chromatin in nuclei that were highly condensed or fragmented. Apoptotic cells also exhibited membrane blebbing. The late apoptotic cells had orange chromatin with nuclei that were highly condensed and fragmented. The necrotic cells had bright orange chromatin in round nuclei. Only cells with yellow, condensed, or fragmented nuclei were counted as apoptotic cells in a blinded, nonbiased manner. 

## 4. Conclusions

Taken together, this work revealed that compounds **4**, **7**, **12b** and **15** may be active cytotoxic agents against different cancer cell lines. This cytotoxic effect was found to be mainly due to apoptosis, which indicated that those compounds may be promising candidate anti-cancer agents, subject to further study. From the chemistry point of view, cytotoxic effect may be due to the presence of free thiol or thioether groups in these compounds.

## 5. Experimental

### 5.1. General

All melting points were taken on an Electrothermal IA 9100 series digital melting point apparatus. The IR spectra (KBr) discs were recorded on a Perkin-Elmer 1650 spectrometer. ^1^H- and ^13^C-NMR spectra were recorded on a Bruker AC-300 Hz instrument. Chemical shifts were expressed as δ (ppm) relative to TMS as internal standard and DMSO-d_6_ as solvent. The elemental analysis were performed at the Micro-analytical Center, Cairo University. Mass spectra were recorded on a Shimadzu GC-MS-QP 1000 EX spectrometer. A domestic microwave oven was used (2450MHz, 800W). The pharmacological study was carried out at the National Research Center (Center of Excellence for Advanced Sciences, Cancer Biology Research Laboratory). All chemicals were from Sigma (NY, USA).

*4-Amino-3-mercapto-6-[2-(2-thienyl)vinyl]-1,2,4-triazin-5(4H)-one* (**1**). *Method A*: a mixture of 2-oxo-4-(2-thienyl)but-3-enoic acid (0.01 mol) and thiocarbohydrazide (0.01 mol) in glacial acetic acid (25 mL) was stirred under reflux for 2 h, cooled to room temperature, and the precipitate that separated was collected by filtration to give yellowish crystals (yield 62%), m.p. 252-255 °C. *Method B*: a mixture of 2-oxo-4-(2-thienyl)but-3-enoic acid (0.01 mol) and thiocarbohydrazide (0.01 mol), were dissolved in a mixture of methylene chloride/methanol (80/20, 15 mL) then silica gel (1.0 g, 200-400 mesh) was added, the solvent was removed by evaporation, and the dried residue was transferred into a glass beaker and irradiated for 1.5-2.0 min in a domestic microwave oven (2450 MHz, 800 W). The product was chromatographed on a silica gel column, using methylene chloride as eluent. Yield 98%, m.p. 254-255 °C. IR (KBr): 3295-3201 cm^−1^ (NH_2_), 1666 cm^−1^ (C=O amide). ^1^H NMR (DMSO-d_6_): δ = 6.51 (s, 2H, NH_2_), 6.74 (d, 1H, *J* = 15.9 Hz, CH=CH_e_), 7.07 (dd appears t, 1H, *J* = 3.60, 4.80 Hz, thiophene-H_b_,) 7.36 (d, 1H, *J* = 3.6 Hz, thiophene-H_c_), 7.57 (d, 1H, *J* = 5.1 Hz, thiophene-H_a_), 7.92 (d, 1H, *J* = 16.0 Hz, CH_d_=CH), 14.00 (s, 1H, SH). ^13^C-NMR (DMSO-d_6_): δ = 118.2, 127.7, 128.3, 128.6, 129.5, 141.0, 141.1, 148.0 and 167.2 (Ar-C, C=C, C=N and C=O). Anal. Calcd for C_9_H_8_N_4_OS_2 _(252.31): C, 42.84; H, 3.20; N, 22.20; Found: C, 42.90; H, 3.16; N, 22.03. MS *m/z* (int. %): 250 (82.8), 251 (90.8), 250 (100), 135 (87.4), 134 (65.5), 69 (80.5), 59 (82.8), 58 (93.1), 51 (75.9), 50 (62.1).

*4-(N-acetylamino)-3-mercapto-5-oxo-6-[2-(2-thienyl)vinyl]-1,2,4-triazine* (**2**). A mixture of 2-oxo-4-(2-thienyl)but-3-enoic acid (0.01 mol) and thiocarbohydrazide (0.01 mol) was dissolved in a mixture of methylene chloride/methanol (80/20, 15 mL) then silica gel (1.0 g, 200-400 mesh) was added, the solvent was removed by evaporation, the dried residue was transferred into a glass beaker and drops of glacial acetic acid were added then the mixture was irradiated for 1.5-2.0 min in a domestic microwave oven (2450 MHz, 800 W). The product was chromatographed on a silica gel column, using methylene chloride as eluent. Yield 85%, m.p. 265-267 °C. IR (KBr): 3202 cm^−1^ (NH), 1667 cm^−1^ (broad, 2 C=O amide). ^1^H-NMR (DMSO-d_6_): δ = 1.91 (s, 3H, CH_3_CO), 6.51 (s, 1H, NH), 6.77 (d, 1H, *J* = 15.9 Hz, CH=CH_e_), 7.11 (dd appears t, 1H, *J* = 3.6 Hz, thiophene-H_b_), 7.41 (d, 1H, *J* = 3.6 Hz, thiophene-H_c_), 7.62 (d, 1H, *J* = 5.1 Hz, thiophene-H_a_), 7.95 (d, 1H, *J* = 16.2 Hz, CH_d_=CH), 14.0 (s, 1H, SH); ^13^C-NMR (DMSO-d_6_): δ = 21.58 (CH3); 121.0, 127.2, 128.0, 128.8, 129.0, 142.3, 143.1, 156.4, 163.6 and 173.7 (Ar-C, C=C, C=N and 2C=O). Anal. Calcd for C_11_H_10_N_4_O_2_S_2_ (294.35): C, 44.88; H, 3.42; N, 19.03; Found: C, 44.90; H, 3.40; N, 19.00. MS *m/z* (int. %): 294 (15.8), 293 (21.1), 253 (47.4), 251 (100), 193 (26.3), 136 (61.8), 135(71.1), 109(32.9), 69 (68.4), 60 (68.4), 58 (90.8).

### 5.2. General Procedure for the Synthesis of Compounds 3, 4 and 5

A mixture of **1** (0.01 mol), oxalyl chloride, chloroacetyl chloride or ethyl chloroformate (0.01 mol) in DMF (15 mL) was refluxed for 6 h, cooled to room temperature and poured onto ice, the precipitate formed separated by filtration and recrystallized from a suitable solvent.

*3-[2-(2-Thienyl)vinyl]-4H,6H-[1,2,4]triazino[3,4-b][1,3,4]thiadiazine-4,7,8-trione* (**3**). Yield 80%, m.p. 338-340 °C, crystallized from DMF/H_2_O. IR (KBr): 3280 cm^−1^ (NH), 1705, 1669 cm^−1^ (C=O and 2 C=O amide). ^1^H-NMR (DMSO-d_6_): δ = 6.82 (d, 1H, *J* = 15.9 Hz, CH=CH_e_), 7.08 (dd appears t, 1H, *J* = 3.9, 4.80 Hz, thiophene-H_b_,) 7.29 (d, 1H, *J* = 3.6 Hz, thiophene-H_c_), 7.51 (d, 1H, *J* = 5.4 Hz, thiophene-H_a_), 8.03 (d, 1H, *J* = 15.9 Hz, CH_d_=CH), 12.31 (s, 1H, NH). ^13^C-NMR (DMSO-d_6_): δ = 120.4, 126.4, 127.5, 128.0, 128.1, 141.6, 141.8, 152.5, 153.6, 159.4 and 179.5 (Ar-C, C=C, 2C=N and 3C=O). Anal. Calcd for C_11_H_6_N_4_O_3_S_2 _(306.32): C, 43.13; H, 1.97; N, 18.29. Found: C, 43.20; H, 1.96; N, 18.35.

*3-[2-(2-Thienyl)vinyl]-4H,6H-[1,2,4]triazino[3,4-b][1,3,4]thiadiazine-4,7(8H)-dione* (**4**). Method 2: A mixture of **1** (0.01mol) and ethyl chloroacetate (0.01 mol) in glacial acetic acid (15 mL) in presence of sodium acetate (0.01 mol) was refluxed for 6 h, cooled to room temperature and poured onto ice, the precipitate formed separated by filtration and crystallized. Yield 72%, m.p. 275-277 °C, crystallized from ethanol. IR (KBr): 3380 cm^−1^ (NH), 1665 cm^−1^ (2C=O, amide). ^1^H-NMR (DMSO-d_6_): δ = 3.82 (s, 2H, SCH_2_), 6.81 (d, 1H, *J* = 15.9 Hz, CH=CH_e_), 7.10 (dd appears t, 1H, *J* = 3.90, 4.80 Hz, thiophene-H_b_), 7.28 (d, 1H, *J* = 3.6 Hz, thiophene-H_c_), 7.52 (d, 1H, *J* = 5.4 Hz, thiophene-H_a_), 8.10 (d, 1H, *J* = 15.9 Hz, CH_d_=CH), 12.35 (s, 1H, NH). Anal. Calcd for C_11_H_8_N_4_O_2_S_2 _(292.33): C, 45.19; H, 2.76; N, 19.17. Found: C, 45.16; H, 2.78; N, 19.15. MS *m/z* (int. %): 292 (66.7), 155 (66.7), 154 (100), 71 (77.8), 66 (66.7), 65 (66.7), 64 (55.6), 61 (88.9).

*3-[2-(2-Thienyl)vinyl]-4H-[1,3,4]thiadiazolo[2,3-c][1,2,4]triazine-4,7(6H)-dione* (**5**). Yield 76%, m.p. 259-261 °C, crystallized from ethanol. IR (KBr): 3295 cm^−1^ (NH), 1675, 1666 cm^−1^ (2C=O, amide). ^1^H-NMR (DMSO-d_6_): δ = 6.50 (br, 1H, NH, thiadiazole), 6.77(d, 1H, *J* = 15.9 Hz, CH=CH_e_), 7.12 (dd appears t, 1H, *J* = 3.90, 4.80 Hz, thiophene-H_b_), 7.42 (d, 1H, *J* = 3.6 Hz, thiophene-H_c_), 7.61 (d, 1H, *J* = 5.4 Hz, thiophene-H_a_), 8.01 (d, 1H, *J* = 15.9 Hz, CH_d_=CH). ^13^C-NMR (DMSO-d_6_): δ = 118.2, 127.8, 128.4, 128.6, 129.6, 141.0, 145.2, 148.1, 165.2 and 167.3 (Ar-C, C=C, 2C=N and 2C=O).Anal. Calcd for C_10_H_6_N_4_O_2_S_2 _(278.31): C, 43.16; H, 2.17; N, 20.13. Found: C, 43.12; H, 2.16; N, 20.23. MS *m/z* (int. %): 278 (12.0), 277 (34.0), 135 (30.0), 121 (44.0), 100 (18.0), 69 (36.0), 58 (20.0), 56 (100).

*8,8-Dimethyl-3-[2-(2-thienyl)vinyl]-8,9-dihydro-4H,6H-[1,2,4]triazino[4,3-b][4,1,2]benzothiadiazine-4,10(7H)-dione* (**6**). A solution of **1** (0.01 mol) and dimedone (0.01 mol) in DMSO (20 mL) and few drops of piperidine was heated under reflux for 24 h, the reaction mixture was cooled, then poured onto ice-cold aq. HCl. The precipitate formed was collected and crystallized from DMF/ethanol (20 mL) to afford a brown powder. Yield 65%, m.p. 280-283 °C. IR (KBr): 3320 cm^−1^ (NH), 1700, 1665 cm^−1^ (2C=O). ^1^H-NMR (DMSO-d_6_): δ =1.25 (s, 6H, 2CH_3_), 2.64 (s, 2H, CH_2_ at C_7_), 3.18 (s, 2H, CH_2_ at C_9_), 6.83 (d, 1H, *J* = 15.9 Hz, CH=CH_e_), 7.12 (dd appears t, 1H, *J* = 3.90, 4.80 Hz, thiophene-H_b_), 7.30 (d, 1H, *J* = 3.6 Hz, thiophene-H_c_), 7.52 (d, 1H, *J* = 5.4 Hz, thiophene-H_a_), 8.11 (d, 1H, *J* = 15.9 Hz, CH_d_=CH), 12.12 (s, 1H, NH). ^13^C-NMR (DMSO-d_6_): δ = 26.80 (2CH_3_), 33.50 (C(CH_3_)_2_), 42.80 (C_9_), 53.5 (C_7_), 95.50, 122.8, 127.5, 128.9, 130.1, 130.8, 141.8, 163.0, 163.5, 163.8, 164.8 and 198.5(Ar-C, C=C, 2C=N and 2C=O).Anal. Calcd for C_17_H_16_N_4_O_2_S_2 _(372.46): C, 54.82; H, 4.33; N, 15.04. Found: C, 54.90; H, 4.23; N, 15.14.

*3-[2-(2-Thienyl)vinyl]-7-thioxo-6,7-dihydro-4H-[1,3,4]thiadiazolo[2,3-c][1,2,4]-triazin-4-one* (**7**). A mixture of **1** (0.01 mol) and CS_2_ (0.01 mol) in ethanol (15 mL) with aq. KOH (5%, 15 mL) was refluxed for 6 h, the reaction mixture was cooled, then poured onto ice-cold aq. HCl. The precipitate formed was collected and crystallized from DMF/ethanol (20 mL) to afford yellow crystals. Yield 63%, m.p. 235-237 °C. IR (KBr): 3285 cm^−1^ (NH), 1668 cm^−1^ (C=O), 1348 cm^−1^ (C=S). ^1^H-NMR (DMSO-d_6_): δ = 6.72 (d, 1H, *J* = 15.9 Hz, CH=CH_e_), 7.10 (dd appears t, 1H, *J* = 3.90, 4.80 Hz, thiophene-H_b_), 7.35 (d, 1H, *J* = 3.6 Hz, thiophene-H_c_), 7.55 (d, 1H, *J* = 5.4 Hz, thiophene-H_a_), 8.01 (d, 1H, *J* = 15.9 Hz, CH_d_=CH), 10.25 (s, 1H, NH). ^13^C-NMR (DMSO-d_6_): δ = 119.3, 126.3, 126.8, 127.7, 128.4, 141.3, 146.1, 147.3, 163.5 and 179.8 (Ar-C, C=C, 2C=N, C=O and C=S). Anal. Calcd for C_10_H_6_N_4_OS_3 _(294.38): C, 40.80; H, 2.05; N, 19.03. Found: C, 40.85; H, 2.10; N, 19.05. MS *m/z* (int. %): 294 (15.0), 252 (96.1), 251 (100), 237 (18.4), 193 (26.3), 162 (21.1), 122(22.4), 109 (32.9), 96 (17.1), 60 (68.4).

*7-Amino-4-oxo-3-[2-(2-thienyl)vinyl]-4,6-dihydropyrazolo[5,1-c][1,2,4]triazine-8-carbonitrile* (**8**). A mixture of **1** (0.01 mol) and malononitrile (0.01 mol) in ethanol (20 mL) with sodium ethoxide (5%, 20 mL) was refluxed for 4 h, the reaction mixture was cooled, then poured onto ice-cold aq. HCl. The precipitate was collected and crystallized from ethanol (20 mL) to afford yellow crystals. Yield 80%, m.p. 247-250 °C. IR (KBr): 3295m^−1^ (NH), 2219 (CN), 1665 cm^−1^ (C=O, amide). ^1^H-NMR (DMSO-d_6_): δ = 6,51 (s, 2H, NH_2_), 6.75 (d, 1H, *J* = 15.9 Hz, CH=CH_e_), 7.10 (dd appears t, 1H, *J* = 3.90, 4.80 Hz, thiophene-H_b_), 7.39 (d, 1H, *J* = 3.6 Hz, thiophene-H_c_), 7.59 (d, 1H, *J* = 5.4 Hz, thiophene-H_a_), 7.99 (d, 1H, *J* = 15.9 Hz, CH_d_=CH), 12.65 (br, 1H, NH). ^13^C-NMR (DMSO-d_6_): δ = 118.2 (CN), 125.5, 127.8, 128.4, 128.6, 129.6, 141.0, 141.2, 148.0, 154.3, 156.8 and 167.3 (Ar-C, C=C, 2C=N and C=O). Anal. Calcd for C_12_H_8_N_6_OS (284.29): C, 50.70; H, 2.84; N, 29.56. Found: C, 50.72; H, 2.90; N, 29.60.

### 5.3. General Procedure for Preparation of 9 and 10

A mixture of **1** (0.01 mol) and dry ammonium thiocyanate (0.01 mol) in glacial acetic acid (20 mL) was refluxed for 3 h, the solid formed on heating was filtered off to give **10** and the filtrate was cooled to room temperature. The precipitate formed was collected and crystallized from ethanol (20 mL) to afford yellow crystals of **9**.

*N-[3-mercapto-5-oxo-6-[2-(2-thienyl)vinyl]-1,2,4-triazin-4(5H)-yl]thiourea* (**9**). Yield 34%, m.p. 240-243 °C. IR (KBr): 3420-3280 cm^−1^ (NH_2_ and NH), 1670 cm^−1^ (C=O amide), 1335 cm^−1^ (C=S). ^1^H-NMR (DMSO-d_6_): δ = 6.28 (br, 2H, NH_2_), 6.72 (d, 1H, *J* = 15.9 Hz, CH=CH_e_), 7.12 (dd appears t, 1H, *J* = 3.90, 4.80 Hz, thiophene-H_b_), 7.40 (d, 1H, *J* = 3.6 Hz, thiophene-H_c_), 7.61 (d, 1H, *J* = 5.40 Hz, thiophene-H_a_), 8.01 (d, 1H, *J* = 15.9 Hz, CH_d_=CH), 12.32 (s, 1H, NH) and 14.12 (s, 1H, SH). ^13^C-NMR (DMSO-d_6_): δ = 118.7, 124.5, 129.3, 138.6, 139.7, 140.7, 148.1, 153.6, 167.4 and 179.8 (Ar-C, C=C, C=o and C=S). Anal. Calcd for C_10_H_9_N_5_OS_3 _(311.40): C, 38.57; H, 2.91; N, 22.49. Found: C, 38.60; H, 2.92; N, 22.49. MS *m/z* (int. %): 311 (1.8), 310 (36.5), 294 (20.3), 277 (28.4), 177 (27.3), 135 (73.0), 122 (24.3), 75 (39.2), 63 (45.9), 57 (100).

*3-[2-(2-Thienyl)vinyl]-7-thioxo-7,8-dihydro[1,2,4]triazolo[5,1-c][1,2,4]triazin-4(6H)-one* (**10**). Yield 42%, m.p. 310-312 °C. IR (KBr): 3228 cm^−1^ (NH), 1665 cm^−1^ (C=O amide), 1328 cm^−1^ (C=S). ^1^H-NMR (DMSO-d_6_): δ = 7.06 (d, 1H, *J* = 15.9 Hz, CH=CH_e_), 7.11 (dd appears t, 1H, *J* = 3.80, 4.80 Hz, thiophene-H_b_), 7.41 (d, 1H, *J* = 3.30 Hz, thiophene-H_c_), 7.66 (d, 1H, *J* = 5.40 Hz, thiophene-H_a_), 8.15 (d, 1H, *J* = 15.9 Hz, CH_d_=CH), 10.27 (s, 1H, NNH), 11.96 (s, 1H, NHCS). Anal. Calcd for C_10_H_7_N_5_OS_2 _(277.32): C, 43.31; H, 2.54; N, 25.25. Found: C, 43.28; H, 2.56; N, 25.22. MS *m/z* (int. %): 277 (34.0), 276 (12.0), 135 (30.0), 121 (44.0), 101 (4.0), 82 (8.0), 69 (36.0), 63 (26.0), 56 (100).

*N-[3-mercapto-5-oxo-6-[2-(2-thienyl)vinyl]-1,2,4-triazin-4(5H)-yl]-N'-phenylthio-urea* (**11**). To a solution of **1** (0.01 mol) in dry dioxane (20 mL) phenyl isothiocyanate was added (0.01 mol) and the reaction mixture was refluxed for 2 h, and the reaction then left to cool to room temperature. The precipitate formed was collected and crystallized from ethanol (20 mL) to afford a yellow solid. Yield 62%, m.p. 263-265 °C. IR (KBr): 3280 cm^−1^ (NH), 1668 cm^−1^ (C=O), 1328 cm^−1^ (C=S). ^1^H-NMR (DMSO-d_6_): δ = 6.77 (d, 1H, *J* = 15.9 Hz, CH=CH_e_), 7.11 (dd appears t, 1H, *J* = 3.90, 4.80 Hz, thiophene-H_b_), 7.22 (d, 1H, *J* = 3.6 Hz, thiophene-H_c_), 7.25-7.62 (m, 5H, Ar-H), 7.67 (d, 1H, *J* = 5.40 Hz, thiophene-H_a_), 7.96 (d, 1H, *J* = 15.9 Hz, CH_d_=CH), 9.76 (s, 1H, NH), 10.9 (s, 1H, NH), 14.02 (s, 1H, SH). ^13^C-NMR (DMSO-d_6_): δ = 118.5, 124.3, 128.3, 128.6, 129.2, 129.5, 138.5, 139.3, 140.9, 141.2, 148.0, 153.5, 167.2 and 179.5 (Ar-C, C=C, C=O and C=S). Anal. Calcd for C_16_H_13_N_5_OS_3 _(387.50): C, 49.59; H, 3.38; N, 18.07. Found: C, 49.60; H, 3.36; N, 18.08.

### 5.4. General Procedure for Preparation of 12_a_ and 12_b_

To a solution of **1** (0.01 mol) in thanol (20 mL) the appropriate aldehyde (0.01 mol) was added followed by HCl (1 mL) and the reaction mixture was refluxed for 2 h, the reaction left to cool to room temperature then poured onto crushed ice and neutralized with dil. ammonium hydroxide. The precipitate formed was collected and crystallized from ethanol (20 mL).

*4-(Benzylideneamino)-3-mercapto-6-[2-(2-thienyl)vinyl]-1,2,4-triazin-5(4H)-one* (**12_a_**). Yield 70%, m.p. 247-250 °C. IR (KBr): 1665 cm^−1^ (C=O, amide). ^1^H-NMR (DMSO-d_6_): δ = 6.80 (d, 1H, *J* = 15.9 Hz, CH=CH_e_), 7.10 (dd appears t, 1H, *J* = 3.90, 4.80 Hz, thiophene-H_b_), 7.41 (d, 1H, *J* = 3.6 Hz, thiophene-H_c_), 7.58-8.00 (m, 5H, Ar-H + thiophene-H_a_ + CH_d_=CH), 8.70 (s, 1H, N=CHPh), 14.12 (br, 1H, sH). ^13^C-NMR (DMSO-d_6_): δ = 118.3, 127.8, 128.4, 128.6, 128.8, 129.1, 129.7, 131.6, 133.1, 141.0, 143.0, 149.1, 169.4 and 173.2 (Ar-C, C=C, C=N and C=O). Anal. Calcd for C_16_H_12_N_4_OS_2 _(340.42): C, 56.45; H, 3.55; N, 16.46. Found: C, 56.51; H, 3.60; N, 16.48.

*3-Mercapto-4-[(2-thienylmethylene)amino]-6-[2-(2-thienyl)vinyl]-1,2,4-triazin-5(4H)-one* (**12_b_**). Yield 76%, m.p. 228-230 °C. IR (KBr): 1665 cm^−1^ (C=O, amide). ^1^H-NMR (DMSO-d_6_): δ = 6.51 (d, 1H, *J* = 15.9 Hz, CH=CH_e_), 6.77 (dd appears t, 1H, *J* = 3.90, 4.80 Hz, thiophene-H_b_), 7.10 (dd appears t, 1H, *J* = 3.90, 4.80 Hz, thiophene-H_b`_), 7.29 (d, 1H, *J* = 3.6 Hz, thiophene-H_c_), 7.41 (d, 1H, *J* = 3.61 Hz, thiophene-H_c`_), 7.62 (d, 1H, *J* = 5.40 Hz, thiophene-H_a_), 7.80 (d, 1H, *J* = 5.42 Hz, thiophene-H_a`_), 7.95 (d, 1H, *J* = 15.9 Hz, CH_d_=CH), 8.05 (d, 1H, *J* = 15.9 Hz, CH_d_=CH), 8.83(s, 1H, N=CH-thienyl), 14.01 (br, 1H, sH). ^13^C-NMR (DMSO-d_6_): δ = 118.2, 127.8, 128.4, 128.6, 129.6, 129.7, 133.4, 135.6, 136.4, 141.2, 143.0, 149.2, 166.7 and 169.5 (Ar-C, C=C, C=N and C=O). Anal. Calcd for C_14_H_10_N_4_OS_3 _(346.45): C, 48.53; H, 2.91; N, 16.17. Found: C, 48.58; H, 2.90; N, 16.18. MS *m/z* (int. %): 346 (14.0), 237 (28.0), 135 (100), 134 (75.3), 121 (46.0), 109 (40.9), 82 (19.4), 58 (40.0), 56 (34.4).

*4-(2,5-Dioxo-1-pyrrolyl)-3-mercapto-6-[2-(2-thienyl)vinyl]-1,2,4-triazin-5-one* (**13**). A mixture of **1** (0.01 mol) and maleic anhydride (0.01 mol), was dissolved in a mixture of methylene chloride/methanol (80/20, 15 mL) then silica gel (1.0 g, 200-400 mesh) was added, the solvent was removed by evaporation. The dried residue was transferred into a glass beaker and irradiated for 1.5-2.0 min in a domestic microwave oven (2450 MHz, 800 W). The product was chromatographed on a silica gel column, using methylene chloride as eluent. Yield 66%, m.p. 330-333 °C. IR (KBr): broad band at 1675-1667 cm^−1^ (3C=O amide). ^1^H-NMR (DMSO-d_6_): δ = 6.75 (d, 1H, *J* = 15.9 Hz, CH=CH_e_), 7.09 (dd appears t, 1H, *J* = 3.60, 4.80 Hz, thiophene-H_b_,) 7.32 (d, 1H, *J* = 3.6 Hz, thiophene-H_c_), 7.53 (d, 1H, *J* = 5.1 Hz, thiophene-H_a_), 7.58 (d, 2H, *J* = 4.5 Hz, pyrrole-H), 7.93 (d, 1H, *J* = 16.0 Hz, CH_d_=CH), 14.08 (s, 1H, SH). ^13^C-NMR (DMSO-d_6_): δ = 118.2, 127.7, 128.4, 128.6, 129.6, 130.2, 141.0, 145.3, 148.0, 149.1 and 167.2 (Ar-C, C=C, C=N and 3C=O). Anal. Calcd for C_13_H_8_N_4_O_3_S_2_ (332.36): C, 46.98, H, 2.43; N, 16.86; Found: C, 46.95; H, 2.40; N, 16.85.

*8-({4-amino-5-oxo-6-[2-(2-thienyl)vinyl]-4,5-dihydro-1,2,4-triazin-3-yl}thio)-3-[2-(2-thienyl)vinyl]-4H,6H-[1,2,4]triazino[3,4-b][1,3,4]thiadiazine-4,7(8H)-dione* (**15**). To a solution of **1** (0.01 mol) in dil. ethanolic KOH (20 mL, 10%) dichloroacetic acid was added (0.01 mol) and the reaction mixture was refluxed for 6 h, then left to cool to room temperature, poured onto ice and HCl. The precipitate formed was collected and crystallized from ethanol (20 mL) to give a yellow solid. Yield 65%, m.p. 195-197 °C. IR (KBr): 3292 cm^−1^ (NH_2_, NH), broad band at1665 cm^−1^ (C=O, amide). ^1^H-NMR (DMSO-d_6_): δ = 3.95 (br, 2H, NH_2_), 4.00 (s, 1H, SCHCO), 6.68 (d, 1H, *J* = 15.9 Hz, CH=CH_e_), 6.73 (d, 1H, *J* = 15.9 Hz, CH=CH_e`_), 7.02 (dd appears t, 1H, *J* = 3.90, 4.80 Hz, thiophene-H_b_), 7.10 (t, 1H, *J* = 3.80, 4.80 Hz, thiophene-H_b`_), 7.39 (d, 1H, *J* = 3.6 Hz, thiophene-H_c_), 7.45 (d, 1H, *J* = 3.60 Hz, thiophene-H_c_), 7.66 (d, 1H, *J* = 5.40 Hz, thiophene-H_a_), 7.82 (d, 1H, *J* = 5.40 Hz, thiophene-H_a_), 7.95 (d, 1H, *J* = 15.9 Hz, CH_d_=CH), 8.0 (d, 1H, *J* = 15.9 Hz, CH_d`_=CH), 13.16 (s, 1H, NH). ^13^C-NMR (DMSO-d_6_): δ = 38.2 (SCHCO), 117.8, 118.1, 118.2, 126.8, 127.7, 128.3, 128.6, 128.7, 129.5, 129.6, 141.0, 141.1, 143.9, 148.0, 152.5, 152.6, 167.1, 172.0 and 172.3 (Ar-C, 2C=C, 4C=N and 3C=O). Anal. Calcd for C_20_H_14_N_8_O_3_S_4_ (542.64): C, 44.27; H, 2.60; N, 20.65; Found: C, 44.23; H, 2.62; N, 20.63. MS *m/z* (int. %): 542 (23.1), 413 (30.8), 320 (61.0), 277 (38.0), 276 (100), 161 (38.5), 108 (38.5), 74 (46).
